# Detection of a rare de novo 18p terminal deletion with inverted duplication in a Chinese pregnant woman

**DOI:** 10.1002/mgg3.868

**Published:** 2019-07-17

**Authors:** Jianjiang Zhu, Hong Qi, Sha Cao, Lirong Cai, Xiaohui Wen, Guodong Tang, Qian Wan, Chen Chen, Juan Wang, Wen Zeng, Yao Luo

**Affiliations:** ^1^ Prenatal Diagnosis Center Beijing Haidian Maternal and Child Health Hospital Beijing P. R. China; ^2^ Annoroad Gene Technology Co. Ltd Beijing P. R. China

**Keywords:** 18p, chromosome recombinant, inverted duplication and terminal deletion, NIPT

## Abstract

**Background:**

The 18p terminal deletion with inverted duplication is an extremely rare chromosome structure abnormality and the common clinical manifestations include intellectual disability and speech delay, etc. Up to now, only three confirmed cases were reported in Europe, and here, for the first time in the Asian population, we report a case of de novo 18p inv‐dup‐del in a Chinese pregnant woman. This structural variation was accidentally discovered by the noninvasive prenatal testing (NIPT) during her prenatal examination.

**Methods:**

Next generation sequencing (NGS) based copy number variations (CNVs) screening and karyotype analysis were performed to verify the type and heredity of the rearrangement, and the fluorescent in situ hybridization (FISH) analysis was also used to confirm the terminal deletion and inverted duplication.

**Results:**

The patient has a de novo 18p11.31‐18p11.1 inverted duplication with a 6.2 Mb 18p terminal deletion. This rare chromosome imbalance, most likely caused by the U‐type exchange mechanism, resulted in the aberrant phenotype of mental disability, speech delay, seizure, and strabismus. However, the rearrangement was not inherited by her unborn child.

**Conclusion:**

This report added a new type of variation to the spectrum of 18p terminal deletion with inverted duplication, and demonstrated that the maternal chromosome rearrangement discovered in NIPT should not just be consider as an interference factor but also a potential indicator of previously undiscovered pathogenic chromosome structure variations in pregnant women.

## 1 INTRODUCTION

The short arm of chromosome 18 is about 15.4 Mb in size and contains around 145 genes (DECIPHER: https://decipher.sanger.ac.uk). The first case of the pathogenic 18p deletion was discovered by the French geneticist Jean de Grouchy in 1963 with clinically defined phenotype (de Grouchy, Lamy, Thieffry, Arthuis, & Salmon, 1963). Since then, at least 150 open‐access patients worldwide with CNV loss in the 18p region have been recorded in the DECIPHER database (DECIPHER: https://decipher.sanger.ac.uk). Moreover, the female‐to‐male ratio is 3:2 for the population affected with total or partial monosomy 18p (Turleau, 2008). The rate of incidence is 1:50,000 live‐born infants (Goyal, Jain, Singhal, & Nandimath, 2017) and 85% of which are thought to be de novo deletions (Spinner, Conlin, Mulchandani, & Emanuel, 2013). Variable degrees of intellectual deficiency and various dysmorphic features are found associated with the disorder. The typical symptoms include growth restriction, abnormalities in limbs, genitalia, brain, eyes and heart, and craniofacial dysmorphism such as round face, dysplastic ears, wide mouth, and dental anomalies. Partial 18p duplication, on the other hand, has been seldom reported due to the extreme rarity. By 2018, only 28 such cases have been described in the literature (Kashevarova et al., 2018), affected patients exhibited a heterogenous array of symptoms and some of which were very similar to those found in partial 18p monosomy. Interestingly, a small fraction of the patients showed a normal phenotype or mild anomalies even when the entire 18p arm is duplicated (Marical et al., 2007). Furthermore, the combination of deletion and duplication on the chromosome 18 short arm is even scarcer and to our knowledge, only six studies from US and Europe have been reported so far.

Noninvasive prenatal testing (NIPT) is one of the most frequently used clinical applications of the next‐generation sequencing (NGS) technology. By utilizing the cell‐free DNA (cfDNA) extracted from the maternal peripheral blood, NGS has demonstrated its predicting power in trisomy 21 screening with high sensitivity and specificity (Snyder et al., 2015). However, the test is not currently considered as a diagnostic method because it still has a small chance of producing false positive or false negative results. One of the several recognized reasons is that the maternal constitutional CNVs could play a crucial role in generating false positive results (Neofytou & Vermeesch, 2018). In a recent systematic review of the literature, maternal CNVs was reported to be responsible for 48% of the false positive trisomy cases (Hartwig, Ambye, Sørensen, & Jørgensen, 2017) and to reduce the influence of maternal CNVs, number of methods such as Digital Analysis of Selected Regions (Kingsley, Wang, & Oliphant, 2015) and feto‐placental chromosome aneuploidy detection (Zhang et al., 2015) were developed. In addition, confined placenta mosaicism, maternal malignancy, vanishing twin, and technical, bioinformatics or human errors were also found to be responsible for the discordance between NIPT‐result and fetal karyotype (Hartwig et al., 2017).

In clinical practice, karyotype and fluorescence in situ hybridization (FISH) are the conventional methods for chromosomal structure variation diagnosis. They could detect chromosomal aneuploidy (gain or loss of chromosomes) and structural aberration with a resolution of 5–10 mega base pairs in size (Rezaei et al., 2018). In recent years, new technologies such as chromosomal microarray analysis (CMA) and NGS based CNV‐seq, with higher resolution and improved detection rate for small CNVs, sparked a revolution in the diagnosis of genetic disorders (Ashoor, Syngelaki, Poon, Rezende, & Nicolaides, 2013). However, CMA and CNV‐seq have limitations in detecting chromosome translocation and inversion, therefore, integrated utilization of multiple genetic testing techniques is prudent to provide reliable results.

In this work, we report, for the first time in Chinese population, a case of de novo 9.2 Mb inverted duplication with a flanking 6.2 Mb terminal deletion on the short arm of chromosome 18 and its associated clinical features. The rearrangement was firstly discovered by NIPT screening and then validated and diagnosed by CNV‐seq, karyotyping, and FISH. Our finding demonstrated that the maternal chromosome rearrangement discovered in NIPT could be utilized as a potential indicator of previously unknown pathogenic chromosome structure variations in pregnant women.

## 2 CASE PRESENTATION

### 2.1 Clinical report

The patient is a 30‐year‐old pregnant woman, gravida 1 para 0, 170 cm and weighted 82 kg at 18 weeks’ gestation. Her initial NIPT result showed an unexpected 5 Mb deletion and 9 Mb duplication on the short arm of chromosome 18. Because of the rare discovery, the patient was then referred to us for genetic counseling sessions and further genetic tests were issued with the complete consent of her parents to investigate if the pregnant woman, her biological parents and the fetus were healthy. After cytogenetic and molecular examinations, a rare de novo 18p terminal deletion with inverted duplication was identified in the pregnant woman, but her parents and the fetus were normal.

The course of her pregnancy was uneventful with the exception of hypothyroidism at 7 weeks’ gestation and treated with Euthyrox from then on. Despite an uneventful family history, the patient had a healthy appearance with slightly poor and slow verbal performance, and she also gave trained answers to certain scenes or questions during genetic counseling sessions rather than engaging herself in independent and creative dialogue. The patient and her family were unaware of the 18p rearrangement. Past medical record only included binocular strabismus as a dysmorphic feature, which was corrected by surgery at the age of 7. Additionally, the patient was discovered to have mild intellectual disability with an intelligence quotient (IQ) value of 78 (evaluated using the Urban version of Chinese Wechsler young children scale of intelligence), and speech delay at the age of 10. Furthermore, according to her parents, she also had perception deficits to stereoscopic structures, some difficulties with mathematics, and a history of epilepsy before adulthood.

The patient has been adequately cared and supported by her family, receiving good education and training since childhood. Currently the patient works full time. Overall, it is difficult to observe any significant abnormality in the patient without close contact or professional examination. Given this, we recommended that her family should continue to take care of the patient, and if she would want to be pregnant again, the prenatal diagnosis or assisted reproductive technology combined with preimplantation genetic testing might be a good option for the birth defect prevention.

## 3 METHOD

### 3.1 NIPT

NIPT screening was performed as previous reported (Yu et al., 2019) at 17 weeks of gestation using the noninvasive prenatal subchromosomal copy number variation detection (NIPSCCD) method, which was low‐pass whole‐genome sequencing based approach.

### 3.2 Karyotype analysis

Karyotyping was carried out on metaphase chromosomes from peripheral blood leukocytes of the patient and her biological parents and amniocyte of the fetus. Chromosomes were harvested according to standard cytogenetic methods and analyzed by G‐bands.

### 3.3 NGS based CNV‐seq

CNV‐seq was performed according to Qi et al (Qi et al., 2018) on the genomic DNA extracted from peripheral blood leukocytes of the patient and amniocyte of the fetus using the Nextseq550AR platform (Annoroad Gene Technology, Beijing, China). It was used to validate and identify the source of the chromosome structure variation detected in the NIPT with a resolution of 100kb.

### 3.4 FISH analysis

The deletion, duplication, and the origin of the chromosome rearrangement on chromosome 18p were verified by fluorescent in situ hybridization (FISH) using the customized BlueFISH bacterial artificial chromosome probes. Samples of peripheral blood leukocytes of the patient and her biological parents were used. The terminal deletion was confirmed by 18pter (18p Subtelomere FISH Probe) green (18p11.3) and 18qter (18q Subtelomere FISH Probe) red (18q23) probes, and the duplicated region was confirmed by the RP11‐297J10 green (18p11.31) and RP11‐463M18 red (18p11.21) probes according to the BlueFish Probes Labeling Protocol (BlueGnome, Cambridge, UK).

## 4 RESULTS

**Figure 1:**
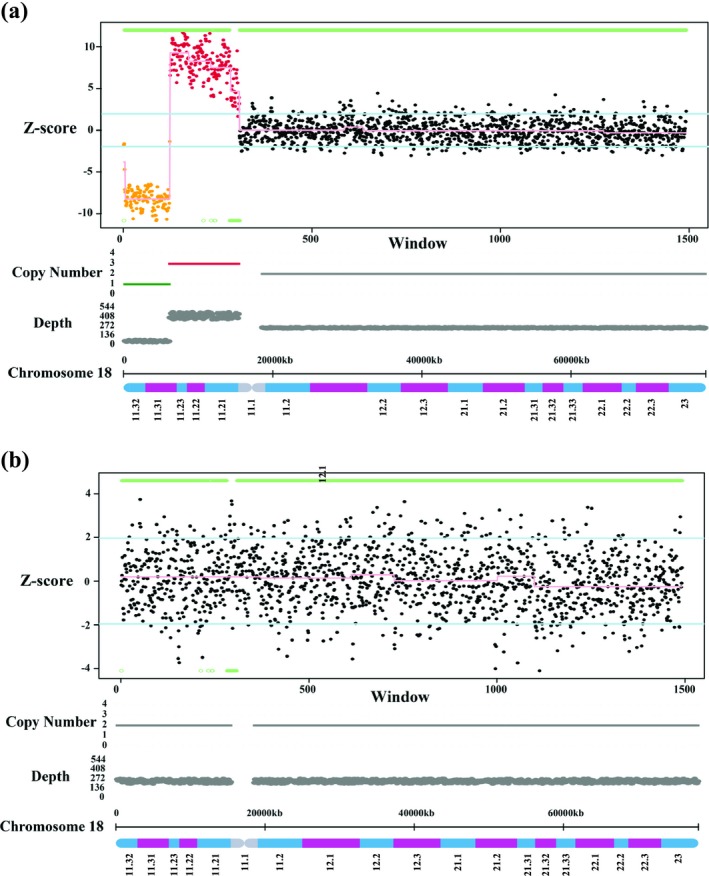
CNV‐seq analysis of the patient and the fetus. (a) CNV‐seq analysis revealed the presence of 18p terminal deletion (green) and duplication (red) in the pregnant woman. (b) CNV‐seq showed the fetus had a normal chromosome18

**Figure 2:**
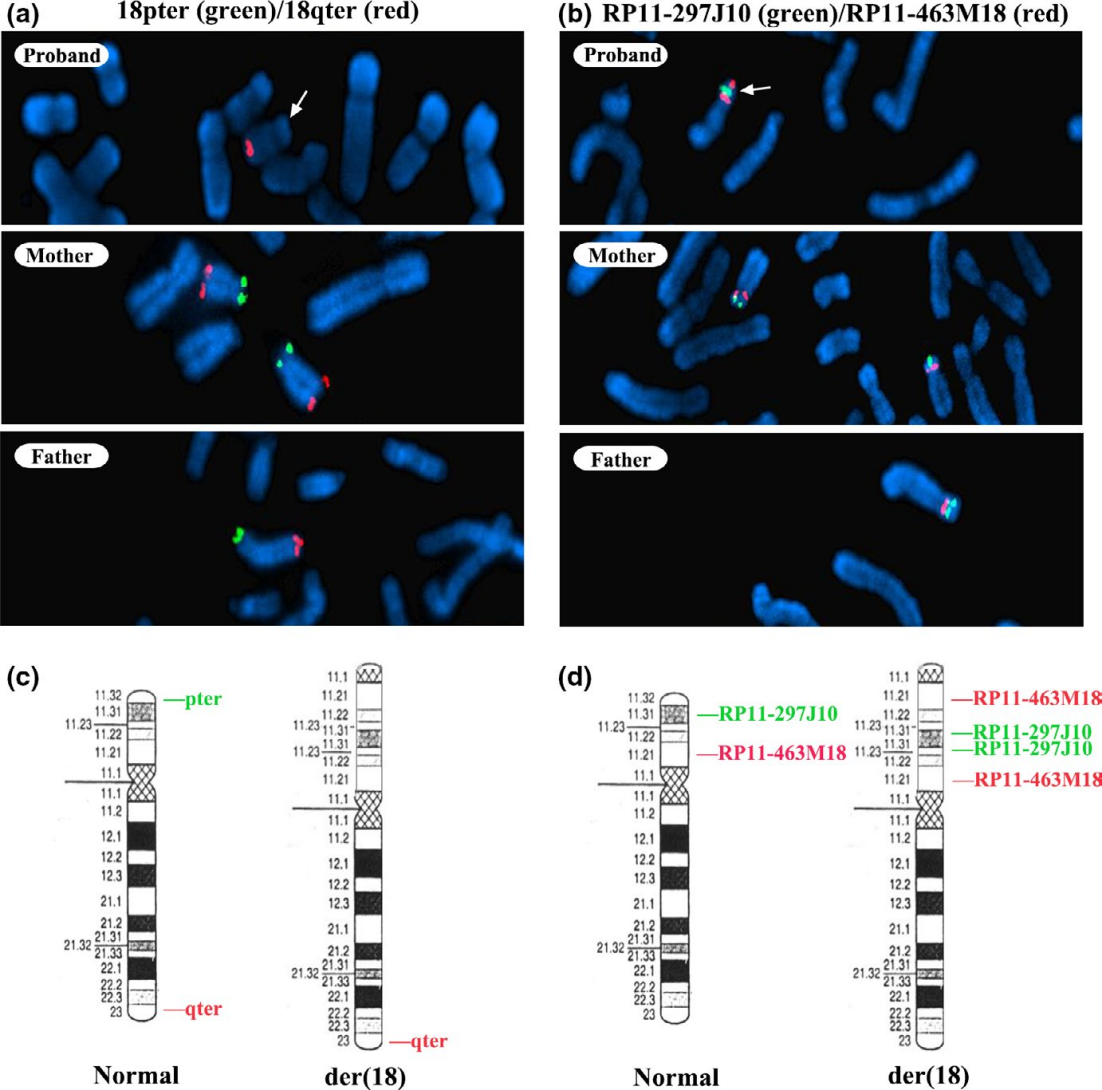
FISH analysis of the patient and her biological parents. (a) The 18p terminal deletion in the patient was confirmed using the 18pter (18p Subtelomere FISH Probe) green (18p11.3) and 18qter (18q Subtelomere FISH Probe) red (18q23) probes, and the parents had normal 18p terminals. (b) The origin of duplication in the patient was determined by RP11‐297J10 green (18p11.31) and RP11‐463M18 red (18p11.21) probes, and the parents did not contain any rearrangements. (c) Idiogram of normal and rearranged chromosome 18 with the corresponding probe positions in (a). (d) Idiogram of the normal and rearranged chromosome 18 with the corresponding probe positions in (b)

After the NIPT screening, a number of genetic tests were carried out to validate the unexpected findings. The karyotype analysis of the patient, her biological parents and the fetus came out uneventful (Figure S1). The NGS‐based CNV‐seq indicated a terminal deletion of 6.2 Mb and a larger duplication of 9.2 Mb on the short arm of chromosome 18 in the proband, but not in her parents nor the fetus (Figure 1). FISH analysis was later performed to find the precise type and origin of the rearrangements. The 18pter (18p Subtelomere FISH Probe) green (18p11.3) and 18qter (18q Subtelomere FISH Probe) red (18q23) probes were used for 18p terminal deletion detection, whereas the RP11‐297J10 green (18p11.31) and RP11‐463M18 red (18p11.21) probes, positioned at the distal to the breakpoint of the two described rearrangements and within the duplication region respectively, were chosen to monitor the 18p duplication. For the terminal deletion detection, both green and red signals were observed on chromosome 18 of the proband's biological parents, but the green signal in the patient was absent. This was a clear indication of terminal deletion in region 18p11.3 (Figure 2). Additionally, the inverted duplication in the patient was confirmed by having two red signals (RP11‐463M18) on either side of the green signal (RP11‐297J10), whereas her parents were normal (Figure 2).

According to all methods used, the proband's karyotype could be written as 46,XX.ish der(18)del(18)(p11.3p11.3)dup(18)(p11.31p11.21)(18pter‐,RP11‐463M18++,RP11‐297J10 enh).seq[GRCh37] der(18)del(18)(p11.32p11.31)dup(18)(p11.31p11.1) chr18:g.[10001_6210000del;6210000_15410000dup].

## 5 DISCUSSION

The terminal deletion accompanied by inverted duplication in the short arm of chromosome 18 is a very rare type of chromosome abnormality. To our knowledge, there are only six literatures mentioned such combined rearrangements in the 18p region, in which only three cases from Europe were confirmed to be the inverted duplication (Table 1). In this study, we are the first to describe a patient containing the 18p inv‐dup‐del in the Chinese population.

**Table 1:** Data of the current and previously reported cases harboring terminal deletion and duplication of 18p

Case number	Reference	Methodology	18p CNV size (Mb)		Inv‐dup	Other CNV	Origin	Age years	Sex	Country	Clinical Assessment
			Del	Dup							
1	Moog et al., 1994	Karyotyping, FISH	2.9	12.5	Yes	NA	De novo	3	Female	Germany	Growth retardation, Speech delay, Hypertonia, Psychomotor retardation, Abnormal EEG, Narrowly arched palate, Epicanthal folds
2	Rossi et al., 2008	aCGH, FISH	0.2	5.4	NA	Del 18q: 12.8 Mb	NA	35	NA	Italy	Growth retardation, Intellectual disability, Asymmetric and broad face, Prominent nasal bridge, Malformed ears, Flexed thumbs, Narrow and tapering fingers, Talipes equinovarus
3			0.5	14.8	NA	Del 18q: 7.8 Mb	NA	5	NA	Italy	Hypertonia, Psychomotor retardation, Abnormal EEG, Facial dysmorphisms, Cleft lip and palate, Vitium cordis
4	Rowe et al., 2009	Karyotyping, FISH, aCGH,	13.5	1.5	NA	NA	De novo	NA	Female	America	NA
5	Misceo et al., 2009	Karyotyping, FISH, aCGH,	7.1	2.3	NA	NA	Maternal inherited	NA	Male	Norway	Intellectual disability, Feeding difficulties, Psychomotor retardation, Microcephaly, Palmar crease, Gastroesophageal reflux
6			7.1	2.3	NA	NA	NA	40	Female	Norway	No symptoms
7	Recalcati et al., 2010	Karyotyping, FISH, aCGH,	2	9.2	Yes	NA	De novo	5	Female	Italy	Short stature, Growth retardation, Speech delay, Motor retardation, Hypertonia, Psychomotor retardation, Epilepsy, Abnormal EEG, Microcephaly, Micrognathia, High‐arched palate, Malformed ears, Low‐set ears, Bulbous nasal tip, Epicanthal folds, Sparse hairs, Blue sclera, Palpebral fissures, Long lashes, Cupid bow lips, Juvenile rheumatoid arthritis, Gastroesophageal reflux
8			10.1	2.6	Yes	Quadruplication 18p: 4.1 Mb	De novo	4	Female	Italy	Short stature, Growth retardation, Speech delay, Malformed ears, Epicanthal folds, Ocular hypertelorism, Long philtrum, Sparse hairs, Retinal hyperpigmentation, Long lashes, Clinodactyly, Palmar crease, Vitium cordis
9	Sireteanu et al. 2013	Karyotyping, SNP array	10.24	1.15	NA	14;18 translocation, dup 16p: 0.5 Mb	De novo	20	Female	Romania	Short stature, Intellectual disability, Speech delay, Behavioral disorders, Muscular hypotonia, Microcephaly, Variable features of the holoprosencephaly spectrum, Triangular face, Blue sclera, Short protruding philtrum/upper lip, Blunted Cupid's bow, Microretrognathia, Preauricular sinus, webbed neck, Broad trunk, Pectus excavatum, Asymmetric mammary glands, Kyphoscoliosis, Wide short hands, Wide short feet, Brachydactyly, Cardiac malformations, Mild hirsutism, Enuresis
our patient		Karyotyping, FISH, NGS	6.2	9.2	Yes	NA	De novo	30	Female	China	Intellectual disability, Speech delay, Epilepsy, Strabismus

Note

aCGH, array‐based comparative genomic hybridization; CNV, copy number variation; Country, the country where the patient was previously identified; Del, deletion; Dup, duplication; FISH, fluorescent in situ hybridization; Inv‐dup, the duplication is inverted or not; NA, not available (feature absent or unrecorded); SNP array, single‐nucleotide polymorphism array; NGS: next‐generation sequencing.

**Figure 3:**
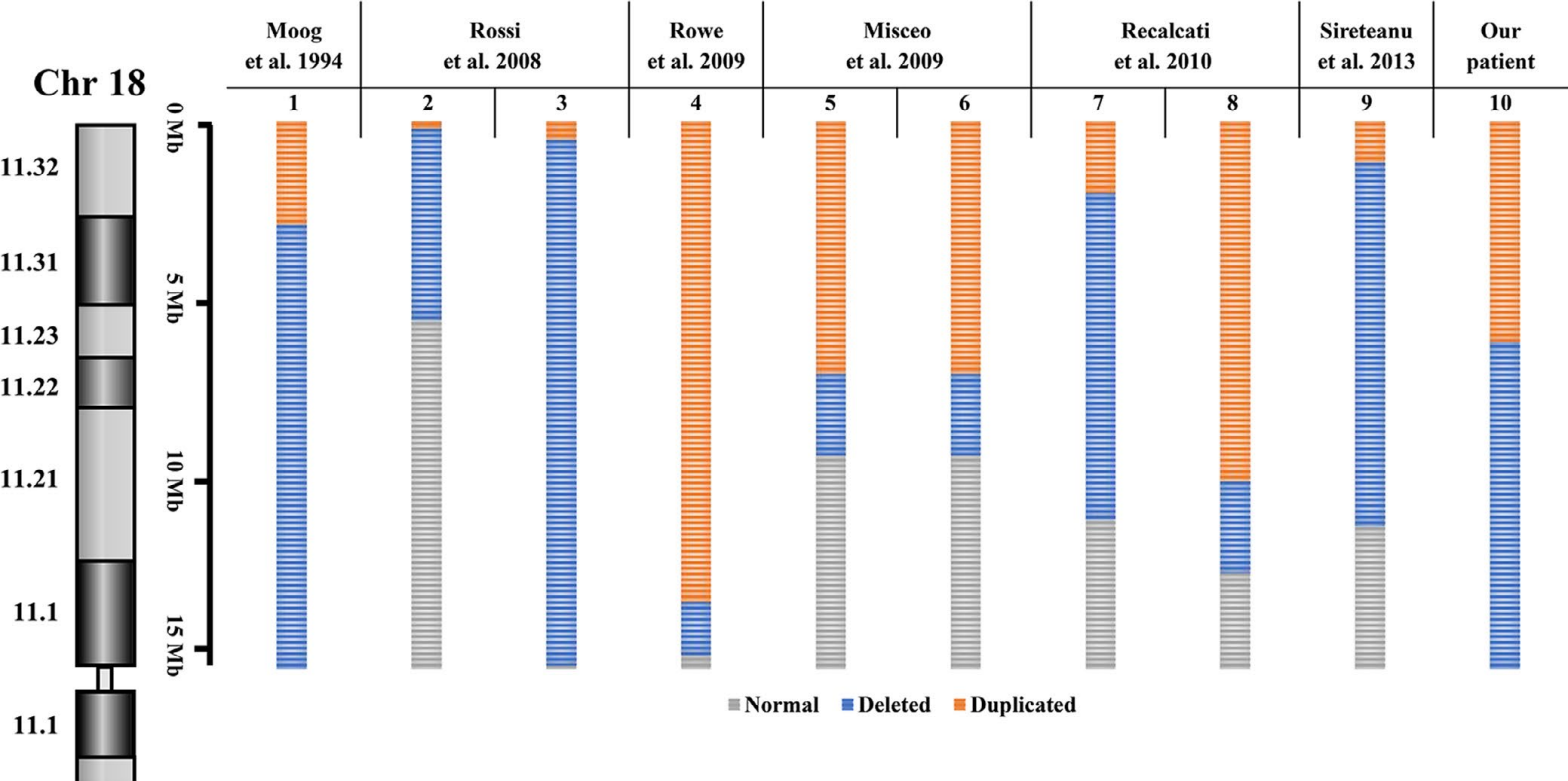
Ideogram representation of our patient in relation to previously reported 18p dup‐del rearrangement cases. Orange lines indicate the deleted segments on chromosome 18p, blue lines demonstrate the duplicated regions in different patients and gray lines represent the normal copy regions on the short arm of chromosome 18

In our case, the deleted region (18p11.32‐18p11.31, 1–6210000) contains 24 protein coding genes. Among them, *LPIN2*, *SMCHD1, *and *TGIF1* are involved in known genetic diseases/ syndromes (DECIPHER: https://decipher.sanger.ac.uk) and the loss of these genes could lead to phenotypes of intellectual disability, delayed speech and language impairment. In addition, there are 41 protein‐related genes located in the reverse duplicated region (18p11.31‐18p11.1, 6210000–15410000), of which *LAMA1*, *MC2R*, and *PIEZO2* are confirmed to cause developmental disorders in multiple unrelated cases. Previously reported patients with duplication in 18p showed moderate intellectual disability, strabismus, and seizure (DECIPHER: https://decipher.sanger.ac.uk). Therefore, either deletion or duplication could potentially lead to the clinical manifestations discovered in our case, it is thus difficult to determine which type of the rearrangements is the primary cause. Also, some phenotypic variabilities between our patient and the aforementioned cases were present and this might be due to the differences in size and location of the rearrangement and the disease penetrance (Figure 3).

In previous literatures, three mechanisms were proposed to explain the origin of the inv‐dup‐del rearrangement. The first mechanism, suggested by Gorinati et al (Gorinati et al., 1991), is based on the fact that either parent carries a paracentric inversion. The second mechanism, on the contrary, relies on the folding, pairing and recombination between sister chromatids within one chromosome (Shaffer & Lupski, 2000). The third mechanism, however, involves a premeiotic double‐strand break with subsequent fusion, or U‐type exchange, between sister chromatids (Weleber, Verma, Kimberling, Fieger, & lubs, H. A., 1976). The second mechanism often associates with the existence of a single copy region between the duplicated and deleted regions on the derivative chromosome, whereas the U‐type exchange does not contain the single copy region and it is thought to be the most frequently seen mechanism for the inv‐dup‐del rearrangement (Rowe et al., 2009). In our case, the NGS data showed a smooth transition between deletion and inverse duplication, so the inv‐dup‐del recombination was most likely caused by the U‐type exchange. However, since the single copy region could be as small as just a couple hundred base pairs and our NGS analysis used 100kb windows, further examination with Sanger sequencing is needed to determine the exact mechanism.

In conclusion, we reported the first case of 18p inv‐dup‐del in the Chinese population and demonstrated that the maternal chromosome rearrangement detected by NIPT during the first trimester of pregnancy should not just be considered as an interference factor, it could also open a gateway to genetic counseling for pregnant women carrying previously unknown de novo or inherited pathological chromosome abnormalities.

## CONFLICT OF INTEREST

The authors declare no conflict of interest.

## AUTHOR CONTRIBUTION

J.Z. clinically reviewed the patient and wrote the manuscript. L.C., X.W., G.T., Q.W., and J.W. performed the experiments and interpreted the data. S.C. and C.C. performed analyses and critically revised the manuscript. W.Z. and Y.L. performed experiments and wrote the paper. H.Q. supervised this project, designed research, analyzed data, and wrote the paper. All authors approved the final version of the manuscript.
