# Supplementary figures and images for: Detection of a rare de novo 18p terminal deletion with inverted duplication in a Chinese pregnant woman
Source: Mol Genet Genomic Med. 2019 Jul 17;7(9):e868. doi: 10.1002/mgg3.868 (PMC6732341; doi:10.1002/mgg3.868)

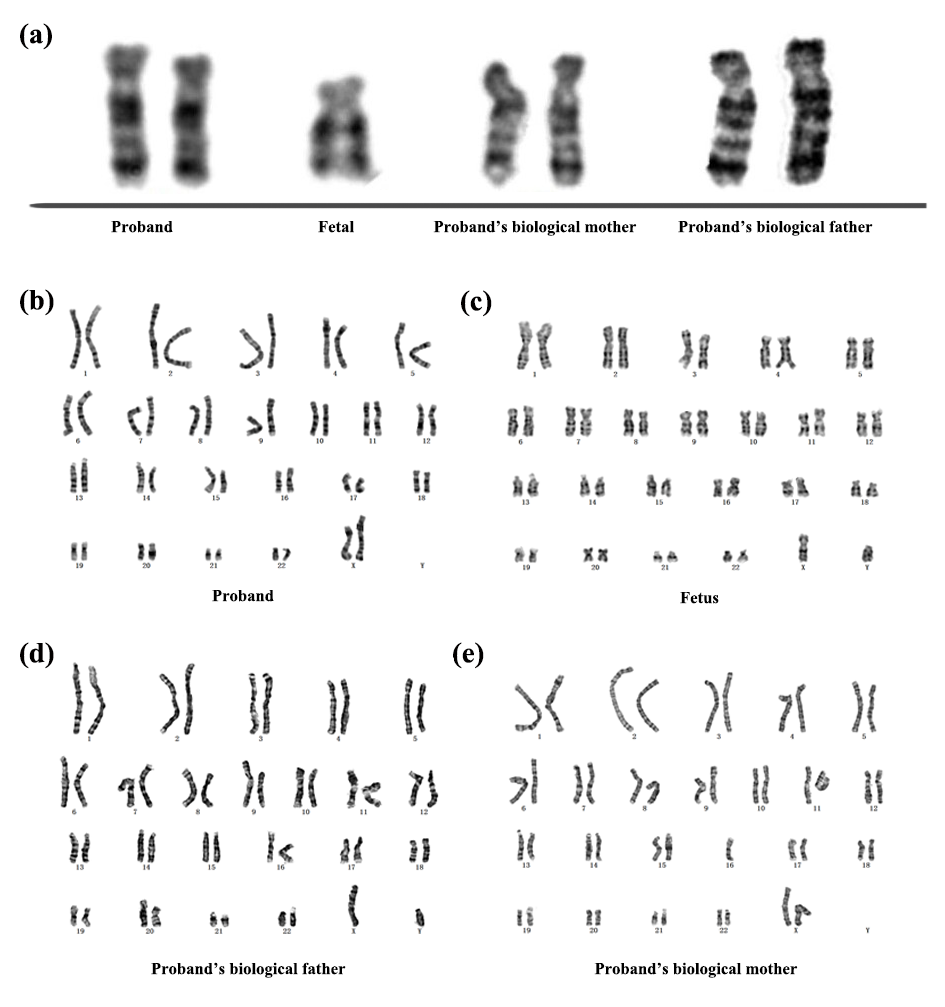

Supplement: Supplementary file 1 [file MGG3-7-e868-s001.tif]
